# Serological Evidence of West Nile and Usutu Viruses Circulation in Domestic and Wild Birds in Wetlands of Mali and Madagascar in 2008

**DOI:** 10.3390/ijerph17061998

**Published:** 2020-03-18

**Authors:** Véronique Chevalier, Maud Marsot, Sophie Molia, Harena Rasamoelina, René Rakotondravao, Miguel Pedrono, Steeve Lowenski, Benoit Durand, Sylvie Lecollinet, Cécile Beck

**Affiliations:** 1 CIRAD, UMR ASTRE, F-34090 Montpellier, France; molia@cirad.fr (S.M.); pedrono@cirad.fr (M.P.); 2 Université Montpellier, F-34090 Montpellier, France; 3 Epidemiology and Public Health Unit, Institut Pasteur du Cambodge, Phnom Penh PO Box 983, Cambodia; 4 University Paris Est, ANSES, Laboratory for Animal Health, Epidemiology Unit, 94700 Maisons-Alfort, France; maud.marsot@anses.fr (M.M.); benoit.durand@anses.fr (B.D.); 5 Centre Régional de Santé Animale, Parc Sotuba, Bamako, Mali; 6 FOFIFA-DRZV, 101 Antananarivo, Madagascar; harena.rasamoelina@coi-ioc.org (H.R.); r.rakotondravao@yahoo.fr (R.R.); 7 UMR 1161 Virology, ANSES, INRA, ENVA, ANSES Animal Health Laboratory, EURL for Equine Diseases, 94704 Maisons-Alfort, France; steeve.lowenski@anses.fr (S.L.); sylvie.lecollinet@anses.fr (S.L.); cecile.beck@anses.fr (C.B.)

**Keywords:** West Nile virus, Usutu virus, seroprevalence, domestic poultry, avifauna, Mali, Madagascar

## Abstract

The geographical distribution and impact on animal and human health of both West Nile and Usutu viruses, two flaviviruses of the Japanese encephalitis complex, have been increasing during the past two decades. Both viruses circulate in Europe and Africa within a natural cycle between wild birds and mosquitoes, mainly from the *Culex* genus. We retrospectively analyzed sera from domestic and wild birds sampled in 2008 in two wetlands, namely the Inner Niger Delta, Mali, and the Lake Alaotra area, Madagascar. Sera were first tested using a commercial ID Screen West Nile Competition Multi-species ELISA kit. Then, positive sera and sera with insufficient volume for testing with ELISA were tested with a Microneutralization Test. In Mali, the observed seroprevalence in domestic birds was 28.5% [24.5; 32.8] _95%CI_, 3.1 % [1.8; 5.2] _95%CI_, 6.2% [3.4; 10.2] _95%CI_ and 9.8 % [7.3; 12.8] _95%CI_, for West Nile virus (WNV), Usutu virus (USUV), undetermined flavivirus, and WNV/USUV respectively. Regarding domestic birds of Madagascar, the observed seroprevalence was 4.4 % [2.1; 7.9]_95%CI_ for WNV, 0.9% [0.1; 3.1] _95%CI_ for USUV, 1.3% [0.5; 2.8] _95%CI_ for undetermined flavivirus, and null for WNV/USUV. Among the 150 wild birds sampled in Madagascar, two fulvous whistling-ducks (*Dendrocygna bicolor*) were positive for WNV and two for an undetermined flavivirus. One white-faced whistling-duck (*Dendrocygna viduata*) and one Hottentot teal (*Spatula hottentota*) were tested positive for USUV. African and European wetlands are linked by wild bird migrations. This first detection of USUV—as well as the confirmed circulation of WNV in domestic birds of two wetlands of Mali and Madagascar—emphasizes the need to improve the surveillance, knowledge of epidemiological patterns, and phylogenetic characteristics of flavivirus in Africa, particularly in areas prone to sustained, intense flavivirus transmission such as wetlands.

## 1. Introduction

West Nile (WNV) and Usutu viruses (USUV) are two zoonotic arboviruses belonging to the Japanese encephalitis virus serocomplex. They present close antigenicity, epidemiology and pathogenesis [1], and both circulate within a natural cycle between wild birds and mosquitoes, mainly from the *Culex* genus. WNV has the most widespread geographic distribution ever described for flaviviruses: it is present in Africa, Europe, Asia, the Middle East, Australia, and Americas [2]. When favorable environmental conditions are met and the epidemiological cycle amplified, the virus may be incidentally transmitted to human and horses and lead to clinical symptoms, ranging from mild fever to fatal encephalitis. Human and horses are considered dead-end hosts [3].

WNV transmission risk results from the local interactions between three components: (i) reservoir hosts, (ii) vectors, both characterized by their diversity, abundance and competence, (iii) and the socio-economic context that impacts the exposure of humans to infectious bites [4]. WNV circulation has been recorded in different ecosystems, including dry or Sahelian areas, urban areas, floodplain forest-meadow ecosystems, and wetlands [5]. However, WNV has historically been associated with wetlands that provide suitable habitats for *Culex* mosquitoes as well as for many wild resident or migrating bird species [6,7,8,9,10]. In France, the majority of West Nile outbreaks occurred in the Camargue region [11]. In Spain, areas close to wetlands (<0.5 km) were found to be suitable for WNV occurrence [12]. In Italy, 91% of the human cases detected between 2008 and 2015 were reported from three regions (Emilia-Romagna, Lombardy and Veneto) located in the Po river plain area [13]. In Romania, areas located in the proximity of the Danube delta were classified at risk [14]. In the Senegal River valley, West Africa, several surveys demonstrated a high level of transmission, either in horses, human, chicken or wild birds [5,15,16,17]. In Madagascar, where WNV lineage 2 was first isolated in 1978 from an endemic parrot species (*Coracopsis vasa*) [18], a recent survey in domestic birds demonstrated an intense circulation of WNV in the Lake Kinkony area, which is the second biggest lake in Madagascar, a stopover site for migrating birds and a refuge for several water fauna including 45 species of water birds [19].

USUV is closely related to WNV. USUV was first detected from mosquitoes in South Africa in 1959 before its detection in many African countries, such as Senegal, Burkina Faso, Ivory Coast, Nigeria, Uganda, Tunisia, Central African Republic, South Africa, and Kenya [20,21,22,23]. In Europe, the virus was reported for the first time in Austria in 2001 but had been identified retrospectively in Italy in 1996 from bird samples [24,25]. Subsequently, the genome of the virus was detected in birds or mosquitoes sampled in the majority of European countries (Belgium, Czech Republic, France, Germany, Hungary, Netherlands, Serbia, Switzerland, and Spain) [26,27,28,29,30,31,32]. Virus ribonucleic acid was also detected in Israel in *Culex* mosquitoes collected in 2014–2015 [33]. USUV can be responsible for high mortalities in wild birds, especially blackbirds (*Turdus merula*) and captive birds belonging to the order Strigiformes (e.g., *Strix nebulosa, Strix aluco, Bubo scandiacus*) [24,34]. USUV is also a zoonotic virus. To date, USUV infection was reported in 19 human patients with meningoencephalitis in Europe [35]. Even if it seems to be less pathogenic to humans than WNV, USUV could become a public health problem in coming years.

Both WNV and USUV geographical distributions have been expanding during the past two decades, with a significant impact either on human and horses, or avifauna health in Europe. Among other reasons, this expansion is due to the spread of both viruses throughout wild bird migrations coming from Africa [36,37,38]. It is well known that both viruses are intensively circulating in Africa, among domestic and wild birds, but a lot remains to be done to better understand their natural history and ecology on the African continent, and to assess the risk that they represent for animal and human health, both in Europe and in Africa.

We retrospectively analyzed sera collected from domestic and wild birds in the framework of an international research project—Ecology and epidemiology of Avian Influenza in developing countries/GRIPAVI (http://gripavi.cirad.fr/en/), dedicated to Avian Influenza (AIV) and Newcastle Disease viruses (NDV) epidemiology in Africa. We estimated the seroprevalence of WNV and USUV for domestic and wild birds sampled in two large African wetlands, i.e., the Mopti area, Mali, located on the Black Sea/Mediterranean wild bird migrating flyway, and the Lake Alaotra region, Madagascar, located on both East Asia/East Africa and on the Black Sea/Mediterranean flyways [39,40].

## 2. Materials and Methods

### 2.1. Study Sites

**Figure 1:**
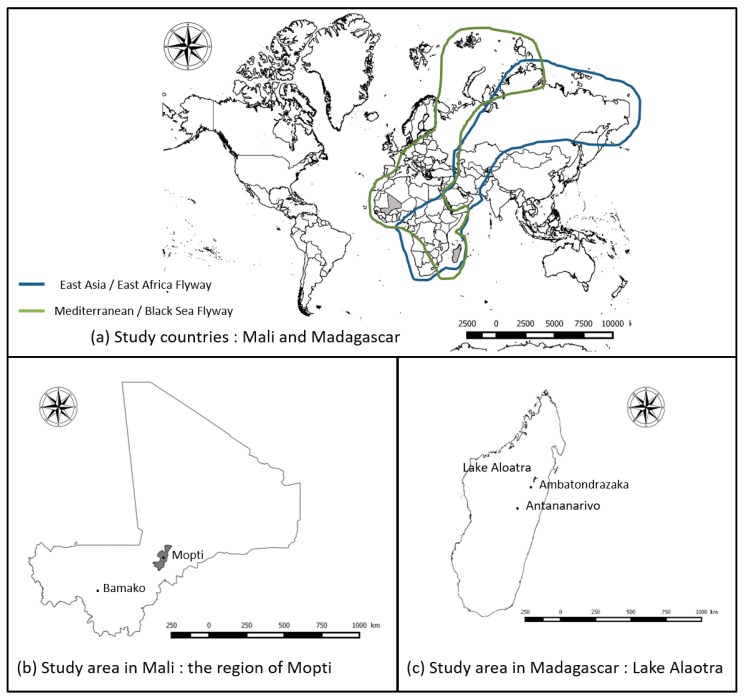
(**a**) Study countries and bird migration pathways (Source: BirdLife International https://www.birdlife.org/), (**b**) study area in Mali: the region of Mopti, and (**c**) study area in Madagascar: Lake Alaotra.

The first study site was the Mopti region in Mali, western Africa (Figure 1a,b). Mopti region is located in the Sahelian zone of Mali and is characterized by a very dry and hot climate, i.e., an annual mean temperature of 28.0 °C, and an annual mean rainfall level of 495 mm. The Mopti region also includes the Inner Niger Delta (IND). IND is the second largest continental wetland in Africa after the Okavango Delta in Botswana, with a maximum flooded area varying between 7000 and 35,000 km^2^ and where millions of Eurasian migratory birds congregate and mix with Afro-tropical water birds remaining in sub-Saharan Africa throughout the year from October to April [41,42,43]. In 2006, according to the Food Agriculture Organization (FAO), most of poultry farms in this region were classified in Sector 4, defined by a low level of biosecurity, animals commonly roaming freely in household backyards, and having frequent contact with other animals including wildlife [44].

The second study site was the Lake Aloatra region in Madagascar (Figure 1a,c). Madagascar is an Indian Ocean island lying 400 km off the eastern coast of South Africa. Lake Aloatra is located in the northern central plateau of the island, near the town of Ambatondrazaka (Figure 1c). This region is characterized by a tropical climate tempered by altitude, with a mean annual temperature of 20 °C and a mean annual rainfall quantity of 1000–1200 mm. It constitutes the largest wetland area of the island with 20,000 hectares (ha) of open water and about 23,000 ha of swamps. It is also the largest rice production basin of Madagascar with 70,000 ha of rice paddies [45]. The Lake Alaotra area was declared a wetland of international importance under the international Ramsar Convention on 2 February 2003. It hosts large populations of water birds. Seventy two wild-bird species have been recorded, of which 50% are present throughout the year [46,47]. Some of these wild birds, in particular Anatidae, such as *Dendrocygna viduata*, *Dendrocygna bicolor*, *Anas melleri* (endemic species in Madagascar), *Anas erythrorhyncha*, *Anas hottentota*, and *Sarkidiornis melanotos*, are intensively hunted by villagers to be sold and consumed. The Lake Alaotra area is also an important goose, duck, and chicken production area: according to Rasamoelina et al., 76% of poultry farms are located close to rice paddies [48].

### 2.2. Data Collection

Samples from Madagascar were collected during a cross-sectional serological survey performed on chickens in a random sample of farms in five municipalities located on the banks of Lake Alaotra. This survey initially aimed at estimating the seroprevalence rates of NDV and AIV in poultry and assessing the link between these seroprevalence rates and potential environmental and farming risk factors. Sample collection was performed in May 2008 [48]. In addition, blood samples (*n* = 150) were opportunistically collected from wild birds trapped by hunters in Lake Alaotra swamps.

In Mali, the same cross-sectional prevalence survey was carried out in May 2008 on chickens and ducks. This period corresponds to the end of the dry-hot season, with an average temperature of 32 °C. The whole protocol design is described in [49]. The study population was the backyard poultry population of villages known to raise ducks by the agents of the Regional Directorates of Veterinary Services of Mopti. Villages and farms were conveniently chosen, based on accessibility criteria and owners’ willingness [49].

In both countries, serum samples were collected by venipuncture in the wing vein on all domestic healthy birds. All birds were resident in the villages without travel history outside the villages. Age and sex of birds was recorded. Birds were considered as juvenile when the farmer reported it was younger than 3 months old. All serum samples were centrifuged in the field, transferred in liquid nitrogen to the laboratory, and stored at −80 °C until analysis.

### 2.3. Laboratory Analyses

When enough volume was available, bird serum samples were tested first using a commercial ID Screen West Nile Competition Multi-species ELISA kit, IDvet, Montpellier, France. Assays were performed according to the method described in Beck et al. [50], and interpreted according to manufacturer’s instructions. This ELISA test uses plates pre-coated with the envelope (E) protein of WNV and measures the competition between antibodies present in the animal serum tested and a monoclonal anti-WNV E antibody conjugated to horseradish peroxidase (HRP). Results were validated if the mean value of the optical density (OD) of the negative control (NC) was > 0.7, and the mean value of the OD of the positive control (PC) out of OD of NC was < 0.3. The threshold value for considering a serum positive was % OD sample (S)/negative control (NC) < 40%. Samples with 40% < % S/NC ≤ 50% were considered as doubtful. Samples with % S/NC > 50% were considered negative. All positive samples by ELISA and all sera with insufficient volume and that could not be tested by ELISA were analyzed by microneutralization tests (MNT) on Vero NK cells, with the WNV lineage 1 IS-98-ST1 and USUV 206795/Italy 2012 strains (kindly provided by Philippe Desprès, IPP and D. Lelli, IZSLER respectively) following the protocol described in Beck et al. [50]. Sera of birds was heat-inactivated at 56 °C for 30 min, then serially diluted (dilution factor of 2, 1/5 to 1/320) and mixed with a volume of 50 µL of Dulbecco’s Modified Eagle’s medium (DMEM), containing 100 tissue culture infectious dose 50 of WNV strain IS-89-ST1, or USUV strain 206795. After incubation at 37 °C for 1.5 h, 2 × 10^4^ Vero cells in 100 µL of DMEM were added to each well. Then, 96-well plates were incubated at 37 °C for 3 days. Microscopic reading was performed to visualize the cytopathogenic effects induced after contact of Vero cells and viruses; its neutralization titer was calculated as the inverse of the latest dilution at which cells were protected. The threshold value for WNV- and USUV-neutralizing antibody titers was 10, as recommended in the Manual of Diagnostic Tests and Vaccines edited by the World Animal Health Organization [51].

Due to serological cross-reactivity between flaviviruses belonging to the Japanese encephalitis virus serocomplex, the MNT was interpreted according to the following rules: (1) if the antibody titer was strictly greater than the other (i.e., positive for only one flavivirus, or titer fourfold greater than the other), the serum was classified against the flavivirus displaying the highest dilution; (2) if the ELISA was positive, but MNT-negative against WNV and USUV, or if the serum was positive for WNV but USUV MNT could not be carried out, the serum was classified as undetermined flavivirus; (3) if the difference in antibody titers was less than fourfold, the serum was classified as WNV/USUV.

### 2.4. Statistical Analysis

Data analyses were performed in R software version 3.4.3 (R Core Team 2018, R: a language and environment for statistical computing, *R Foundation for Statistical Computing, Vienna, Austria. ISBN 3-900051-07-0, URL* http://www.R-project.org/) [52], and *p* ≤ 0.05 denoted statistical significance. Seroprevalence rates and associated confidence intervals at 95% were computed for domestic birds in Madagascar and Mali. Then, when the information was available for domestic birds, the seroprevalence rates (with the 95% confidence intervals) according to age, sex, and species were computed. Due to the very low level of infection for WNV and USUV of wild birds collected in Madagascar, only the number of positive wild birds for flavivirus, WNV and USUV were presented. To compare the seroprevalence rates of domestic birds between Madagascar and Mali, and within a country between (i) female and male, (ii) adult and juvenile birds, and (iii) bird species, Chi-square tests were applied (or Fisher’s tests if the assumptions for Chi-square tests were not satisfied). If significant, the corresponding seroprevalence rates per category (age, sex, or species) were compared to identify the most exposed category of birds.

## 3. Results

### Study Population

From the initial number of blood samples, a total of 702 domestic bird samples was available for WNV and USUV analyses: 232 in Madagascar, and 470 in Mali. The number of sampled domestic birds by age, sex, and species is shown in Table 1. The majority of domestic birds sampled were females and adults in both countries. Only chickens were sampled in Madagascar, whereas the same number of ducks and chicken were collected in Mali (*n* = 235, Table 1).

A total of 150 wild birds were sampled in Madagascar, belonging to 8 different species (white-faced whistling-duck *n* = 63, fulvous whistling-duck *n* = 43, red-billed teal *n* = 14, Hottentot teal *n* = 10, common moorhen *n* = 10, Meller’s duck *n* = 7, African comb duck *n* = 2 and Madagascan rail *n* = 1).

The numbers of tested birds by ELISA and positive by ELISA are shown in Table 2a. The positive sera by ELISA and the non-tested samples by ELISA were tested by MNT. The associated results, i.e., the number of birds positive for WNV, USUV, undetermined flavivirus, or WNV/USUV by MNT are shown in Table 2b.

**Figure 2:**
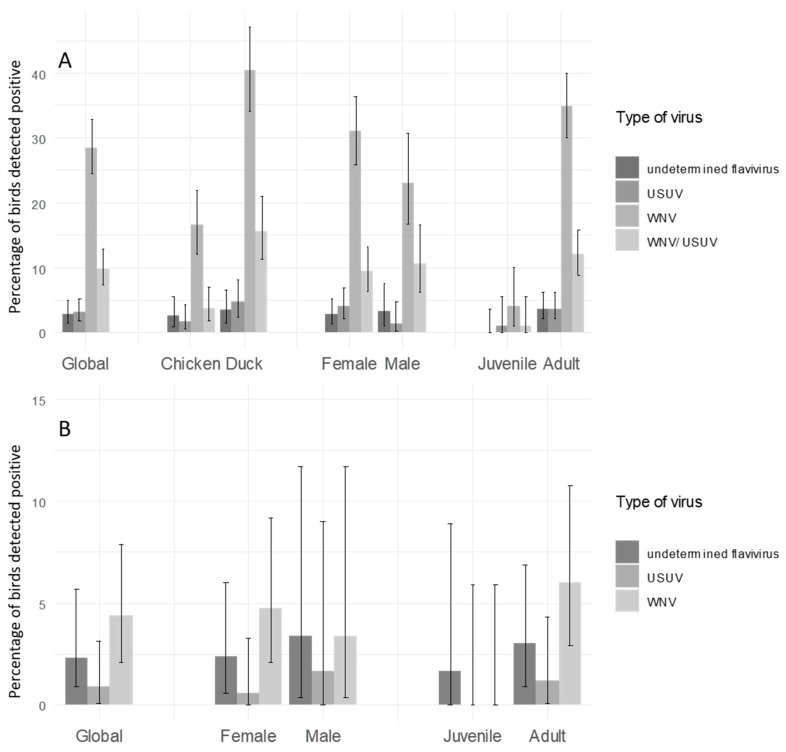
Seroprevalence of West-Nile and Usutu viruses in domestic birds. (**A**) in the region of Mopti in Mali (**B**) in Madagascar. The y-axis represents the percentage of birds detected positive for West Nile virus (WNV), Usutu virus (USUV), West Nile and Usutu viruses (WNV/USUV) and undetermined flavivirus.

In domestic birds, the overall seroprevalence for WNV, USUV, WNV/USUV, and for an undetermined flavivirus, and the associated confidence intervals in the two study sites, are given in Figure 2. In Mali, the observed seroprevalence in domestic birds was 28.5% [24.5; 32.8] _95%CI_, 3.1 % [1.8; 5.2] _95%CI_, 6.2% [3.4; 10.2] _95%CI_ and 9.8 % [7.3; 12.8] _95%CI_, for WNV, USUV, undetermined flavivirus, and WNV/USUV respectively. Regarding domestic birds of Madagascar, the observed seroprevalence was 4.4 % [2.1; 7.9]_95%CI_, 0.9% [0.1; 3.1] _95%CI_, 1.3% [0.5; 2.8] _95%CI_ and null, for WNV, USUV, undetermined flavivirus, and WNV/USUV respectively. The associated confidence intervals of seroprevalence rates by category of birds (age, sex, and bird species) are also presented in Figure 2.

Six wild birds among 150 birds sampled detected positive in Madagascar (Table 3). Two fulvous whistling-ducks were positive for WNV (seroprevalence 1.3% [0.2;4.7] _95%CI_, and two for an undetermined flavivirus (one by ELISA and the other one by MNT, Table 3). One white-faced whistling-duck and one Hottentot teal tested positive for USUV (Table 3).

According to Chi-square tests (or Fisher’s tests if the assumptions for Chi-square tests were not satisfied), the seroprevalence rate in domestic birds was significantly higher in Mali than in Madagascar (Figure 2). In Madagascar, there was no significant difference of seroprevalence rates between ages age (*p*-value = 0.19) and sex (*p*-value = 0.73) (Figure 2). In Mali, WNV and WNV/USUV seroprevalence rates were significantly higher in ducks than in chicken (*p*-value < 0.001). Juvenile birds showed a lower seroprevalence than adults.

## 4. Discussion

To our knowledge, this survey constitutes the first evidence of WNV and USUV circulation in the IND, Mali, and in the Lake Alaotra region, Madagascar—and the first estimation of WNV and USUV seroprevalence in domestic and/or wild birds in these regions. Several studies have already highlighted that WNV was endemic in Madagascar [53], with an antibody prevalence of 46.2% ([37.4–55.2%]_95%CI_) in horses sampled in 2010 in the Highlands [54], and of around 20% in the human population, mainly sampled in and around Antananarivo, the capital city of Madagascar [55]. Our results clearly show that WNV is circulating in the Lake Alaotra region. The low observed seroprevalence in birds (4.4%) is in agreement with previous observations, with a more intense circulation in the warm and dry areas than in the cool Highlands [53]. In comparison with other countries, we found an antibody prevalence in domestic birds lower than the one estimated in Kerala, India (11,5%; *n* = 209) [56], in the range of what has been estimated in Greece (4.2% of 240 sampled chickens) [57], and higher than that was previously found in the Lake Kinkony region, Madagascar (0.2% in ducks and chickens) [19]. However, the climatic and ecological contexts—as well as the sampling period and sizes—are so different that a comparison between these three estimations would not be relevant.

Until now, only two wild bird genera were found WNV-positive in Madagascar, i.e., *Egretta* sp. and *Coracopsis* sp. [58]. In our survey, only one species—*Dendrocygna bicolor—*tested positive (2/43). The estimated prevalence rate in wild birds (1.3%) is in accordance with results from South Korea (1.6%) and Japan (1.1%) [59,60], but lower than the seroprevalence (5.5%, *n* = 422) estimated in 2003 in northern Senegal [16]. Although mallards and other waterfowl are presumed to be less permissive than domestic bird species, experimental infection studies have shown that mallards (*Anas platyrhynchos*) and aigamo ducks (*Anas platyrhynchos var. domesticus*), a cross between wild and domestic ducks, are susceptible to WNV infection, and develop viremia titers sufficient to infect mosquitoes [61]. Passerines are generally considered to play a major role in WNV circulation [62,63]. However, water birds may also be involved and their role in WNV circulation and long-distance spread needs to be better assessed.

WNV is circulating in Madagascar, and despite the presence of 29 mosquito species associated with WNV transmission, neither outbreak in humans nor in animals has been reported [64]. The island displays specificities regarding WNV eco-epidemiology which may help to explain why WNV circulates less actively in Madagascar than in sub-Saharan Africa. First, the avifauna biodiversity is low, with 282 known taxa as compared to 1 046 reported from Tanzania for instance [65], and nothing is known about the host-competency of these birds for WNV. Second, the island is not located within the main migratory route of breeding Palearctic birds, water birds or passerines, and less than five Palearctic migrating passerines species have been documented on the island [53]. Lastly, the WNV lineage 2 strain isolated in Madagascar forms a separate sub-clade from sequences obtained from African and Eurasian cases [66]. As already mentioned by Tantely et al., Malagasy specificities, combined with the huge eco-climatic diversity of the island suggest a very specific WNV eco-epidemiological context that remains to be explored [53]. The occurrence of one fatal clinical case reported in 2011—of a 58-year-old woman coming back from Madagascar [67]—justifies the need for further investigations into the role of ecological and climatic drivers in WNV circulation in this country and an improved identification of areas and periods at risk.

Several serological surveys implemented in West Africa showed an endemic circulation of WNV [5,15,16,68,69,70]. Despite our results showing a higher prevalence in domestic birds in Mali than in Madagascar, no human cases were reported. Absence of human cases in Mali may be due to misreporting, underreporting, or limited human exposure. In any case, given the high prevalence found in domestic birds, WNV circulation should be monitored. A single publication reports very high anti-WNV IgG prevalence rates (31–43%) in 376 serum samples collected between 2009 and 2013 from acutely ill human patients [71]. Our results confirm that WNV is circulating in the IND region, located on the Black Sea/Mediterranean wild bird migrating flyway (Figure 1). Observed prevalence was higher in ducks than in chickens. This result may be explained by a longer life expectancy in ducks. However, mosquito feeding behavior may also partially contribute to this result. A study of host-feeding patterns in *Culex* mosquitoes performed in Iran showed that ducks (*Anas spp.*) were among the four most commonly detected hosts in blood meals from the four most abundant mosquito species, i.e., *Culex pipiens* form *pipiens*, *C. theileri*, *C. sitiens,* and *C. perexiguus*, all potential vectors of WNV [72]. Several surveys implemented in different continents suggest the involvement of ducks in WNV epidemiological cycle, either as amplifying hosts or as reservoirs. Neutralizing antibodies were found in 43 of 423 domestic ducks sampled in Turkey between 2011 and 2013 [73]. In 2005, an outbreak in a commercial flock induced significant mortalities and high prevalence rates in exotic and domestic ducks [74]. Even if the existence of bird-to-bird direct transmission could partially explain this outbreak [62,75,76], further investigations are needed (i) to assess the role of ducks, particularly in areas of high densities, such as Madagascar Highlands or in Southeast Asia, and (ii) to infer their potential use in endemic and at-risk areas for WNV surveillance.

Our results provide the first evidence of USUV circulation in Mali and in Madagascar, with a higher seroprevalence rate in domestic birds in Mali than in Madagascar (3.1% vs. 0.9%). There was no earlier USUV serology or isolation record in Madagascar, but USUV specific-antibodies were already detected in the Indian Ocean region from seabirds [77]. In West Africa, USUV was isolated during arbovirus surveillance program in neighboring Senegal, from 10 pools of *C. neavei* and 2 pools of *C. antennatus* collected near four temporary ponds [78]. Mali is located in the Black Sea/Mediterranean flyway (Figure 1), and as demonstrated by Engel et al., migrating birds may regularly introduce the virus from the IND to Europe [36].

Due to a particularly high diversity and abundance of wild birds and suitable habitats for *Culex* mosquitoes, wetlands are known to facilitate flavivirus circulation. Even if IND and Lake Alaotra differ to some extent, i.e., climate, elevation, and their locations are on two different bird migration pathways, both regions sustain WNV and USUV circulation. As demonstrated in other countries, our results also show that both viruses co-circulate in Mali [79].

According to Engel et al., USUV was introduced regularly from Africa into Europe in the last 50 year. Authors also showed that the genetic diversity of European lineages was primarily driven by virus evolution in Africa [36]. Wild bird migrations also transport WNV between Africa and Europe [38]. Due to environmental changes—climate, landscape, and land use, as well as a global rise of travel and good trade—frequency, and severity of WNV and USUV outbreaks, as well as their respective geographic distributions, increased in the last two decades in Europe [79]. How the transmission of both viruses and the risk of outbreaks will evolve in Africa is unknown. Therefore, there is a need to improve the surveillance and our knowledge of flavivirus epidemiological patterns as well as phylogenetic characteristics in Africa, particularly in areas prone to sustained, intense flavivirus transmission, such as wetlands.

## 5. Conclusions

We retrospectively analyzed sera from domestic and wild birds sampled in 2008 in two wetlands, namely the Inner Niger Delta, Mali, and the Lake Alaotra area, Madagascar. In Mali, the observed seroprevalence in domestic birds was 28.5% [24.5; 32.8] _95%CI_, 3.1 % [1.8; 5.2] _95%CI_, 6.2% [3.4; 10.2] _95%CI_ and 9.8 % [7.3; 12.8] _95%CI_, for West Nile virus (WNV), Usutu virus (USUV), undetermined flavivirus, and WNV/USUV respectively. Regarding domestic birds of Madagascar, the observed seroprevalence was 4.4 % [2.1; 7.9]_95%CI_ for WNV, 0.9% [0.1; 3.1] _95%CI_ for USUV, 1.3% [0.5; 2.8] _95%CI_ for undetermined flavivirus, and null for WNV/USUV. Among the 150 wild birds sampled in Madagascar, two fulvous whistling-ducks (*Dendrocygna bicolor*) were positive for WNV and two for an undetermined flavivirus. One white-faced whistling-duck (*Dendrocygna viduata*) and one Hottentot teal (*Spatula hottentota*) were tested positive for USUV. African and European wetlands are linked by wild bird migrations. This first detection of USUV—as well as the confirmed circulation of WNV in domestic birds of two wetlands of Mali and Madagascar—emphasizes the need to improve the surveillance, knowledge of epidemiological patterns, and phylogenetic characteristics of flavivirus in Africa, particularly in areas prone to sustained, intense flavivirus transmission such as wetlands.

## Figures and Tables

**Table 1:** Number of domestic birds sampled by sex, age and species in Madagascar and Mali.

Country	Samples	Female	Male	Juvenile	Adult	Chicken	Duck
Madagascar	232 *	169	60	63	166	232	-
Mali	470	319	151	98	372	235	235

* 3 domestic birds were not sexed and aged.

**Table 2:** (**a**) Number of birds sampled, tested by ELISA, and detected positive by ELISA in Madagascar (domestic and wild birds) and in Mali (domestic birds); (**b**) Number of birds tested by microneutralization tests (MNT) and detected positive for West Nile virus (WNV), Usutu virus (USUV), undetermined flavivirus or for WNV/USUV in Madagascar (domestic and wild birds) and in Mali (domestic birds).

**(a) ELISA**							
**Country**	**Type of Birds**	**ELISA**					
		**Tested**			**Positive**		
Madagascar	domestic birds	195			4		
	wild birds	64			1		
Mali	domestic birds	457			199		
**(b) MNT**							
**Country**	**Type of Birds**	**MNT**					
		**Tested**	**WNV**	**USUV**		**Flavivirus**	**WNV/USUV**
Madagascar	domestic birds	41 *	10	2		6	0
	wild birds	87 *	2	2		2	0
Mali	domestic birds	212 *	134	15		14	46

* sera tested by MNT are all sera positive sera by ELISA and sera with insufficient volume that could not be tested by ELISA, i.e., 86 wild birds and 37 domestic birds in Madagascar, and 13 domestic birds in Mali.

**Table 3:** Species, positivity for WNV, USUV or undetermined flavivirus, and results associated with ELISA (%S/N), MNT (titer) for the 6 wild birds detected positive in Madagascar.

Sample ID	Species	Positivity	ELISA	%S/N	MNT	Titer	USUV	Titer
1	*Fulvous whistling-duck*	WNV	-	-	positive	160–320	negative	-
2	*Fulvous whistling-duck*	WNV	-	-	positive	10–20	negative	-
3	*Fulvous whistling-duck*	flavivirus	-	-	positive	160	not tested	-
4	*Fulvous whistling-duck*	flavivirus	positive	39	negative	-	negative	-
5	*White-faced whistling-duck*	USUV	-	-	negative	-	positive	10
6	*Hottentot teal*	USUV	-	-	positive	10	positive	80
